# Anti‐human CD9 antibody Fab fragment impairs the internalization of extracellular vesicles and the nuclear transfer of their cargo proteins

**DOI:** 10.1111/jcmm.14334

**Published:** 2019-04-13

**Authors:** Mark F. Santos, Germana Rappa, Jana Karbanová, Cheryl Vanier, Chikao Morimoto, Denis Corbeil, Aurelio Lorico

**Affiliations:** ^1^ College of Medicine Touro University Nevada Henderson Nevada; ^2^ Biotechnology Center and Center for Molecular and Cellular Bioengineering Technische Universität Dresden Dresden Germany; ^3^ Department of Therapy Development and Innovation for Immune Disorders and Cancers Graduate School of Medicine Juntendo University Bunkyo‐ku Tokyo Japan; ^4^ Mediterranean Institute of Oncology Viagrande Italy

**Keywords:** cancer, CD9, endocytosis, extracellular vesicle, Fab fragment, nucleoplasm

## Abstract

The intercellular communication mediated by extracellular vesicles (EVs) has gained international interest during the last decade. Interfering with the mechanisms regulating this cellular process might find application particularly in oncology where cancer cell‐derived EVs play a role in tumour microenvironment transformation. Although several mechanisms were ascribed to explain the internalization of EVs, little is our knowledge about the fate of their cargos, which are crucial to mediate their function. We recently demonstrated a new intracellular pathway in which a fraction of endocytosed EV‐associated proteins is transported into the nucleoplasm of the host cell via a subpopulation of late endosomes penetrating into the nucleoplasmic reticulum. Silencing tetraspanin CD9 both in EVs and recipient cells strongly decreased the endocytosis of EVs and abolished the nuclear transfer of their cargos. Here, we investigated whether monovalent Fab fragments derived from 5H9 anti‐CD9 monoclonal antibody (referred hereafter as CD9 Fab) interfered with these cellular processes. To monitor the intracellular transport of proteins, we used fluorescent EVs containing CD9‐green fluorescent protein fusion protein and various melanoma cell lines and bone marrow‐derived mesenchymal stromal cells as recipient cells. Interestingly, CD9 Fab considerably reduced EV uptake and the nuclear transfer of their proteins in all examined cells. In contrast, the divalent CD9 antibody stimulated both events. By impeding intercellular communication in the tumour microenvironment, CD9 Fab‐mediated inhibition of EV uptake, combined with direct targeting of cancerous cells could lead to the development of novel anti‐melanoma therapeutic strategies.

## 1 INTRODUCTION

Growing evidence indicate that intercellular communication in multicellular organisms is mediated not only by direct cell‐cell contact or soluble molecules, but also by extracellular vesicles (EVs), ie lipid bilayer‐enclosed nanobiological units actively released from all cell types.^1^, ^2^ In contrast to soluble signalling molecules, bioactive compounds associated with EVs (eg, proteins, nucleic acids such as non‐coding RNA [including microRNA], mRNA and genomic DNA) are protected from degradation.^3^, ^4^ EVs are found in internal and external bodily fluids and act as mediators of long‐distance transfer of biological information. Physiological and pathological conditions determine the nature of EVs released by the producing cells as well as the abundance of their bioactive cargo molecules.^5^ Under physiological states, EVs can play important roles during embryonic development and afterward in the homoeostasis of various organ systems (reviewed in Ref.^6^). In cancer, they could promote pro‐angiogenic events and alter the surrounding cellular components as well as extracellular matrix to develop the pre‐metastatic niche.^7^, ^8^ With regard to clinical purposes, EVs attract additional interest because their production is deregulated in human diseases, notably in cancer; hence, their cargo molecules can be monitored as biofluid‐associated markers.^9^, ^10^ Furthermore, EVs can be engineered for the selective therapeutic delivery of biomacromolecules.^6^, ^11^

Two major general pathways were ascribed to explain the biogenesis and release of EVs by donor cells as exosomes or ectosomes.^12^ The first class of EVs is derived from the internal intraluminal vesicles of multivesicular bodies (MVBs) that are formed by the inward budding of the endosomal membrane during the maturation of MVBs. Upon fusion with the plasma membrane, MVBs release them outside the cell. The diameter of exosomes varies from 30 to 120 nm. Outward budding and fission of plasma membrane generate the second class of EV. Thus, ectosomes are typically larger than exosomes and their diameter varies from 100 nm to 1 μm. We have previously shown that ectosomes can bud from microvilli and/or cilia.^13^, ^14^ Once released into the extracellular milieu, the uptake of EVs by recipient cells can be accomplished by several molecular mechanisms of internalization, which are not mutually exclusive,^15^, ^16^, ^17^, ^18^ such as clathrin‐mediated endocytosis^17^ or lipid raft‐dependent endocytosis.^19^ In spite of this knowledge, fundamental questions remain about the fate of endocytosed EVs particularly their biological cargo, which is crucial for their function.^20^

**Figure 1:**
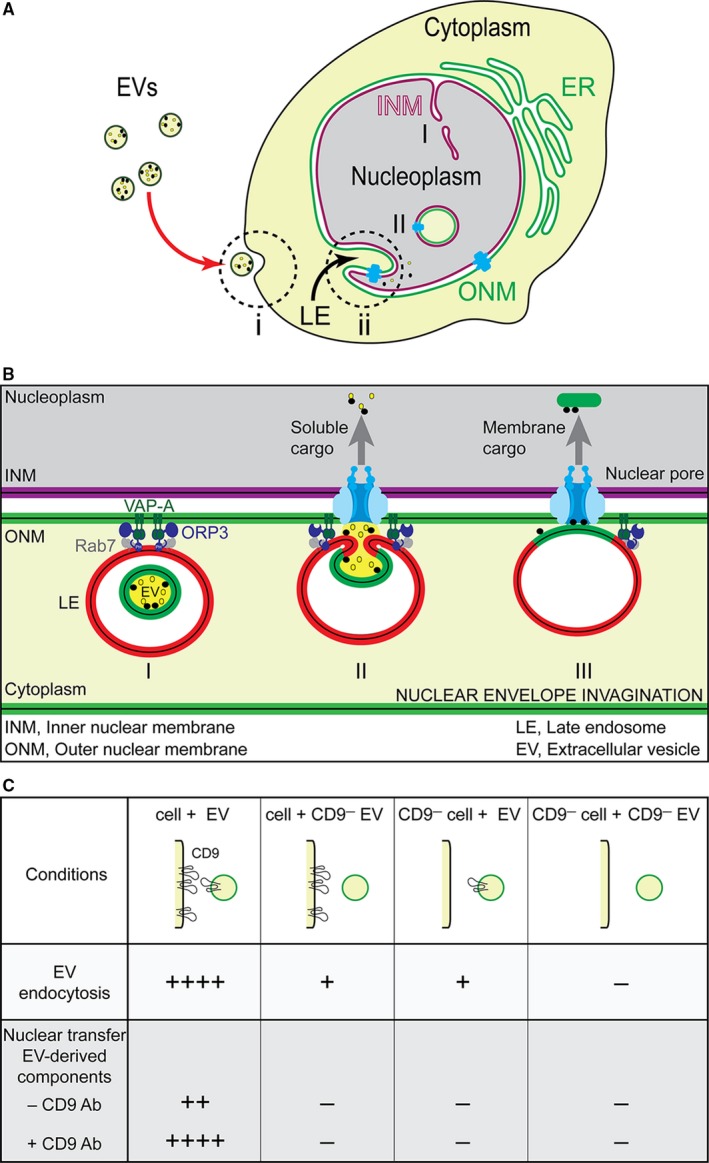
Entry and delivery of extracellular vesicles (EV)‐derived cargo molecules into the nucleoplasm of recipient cells. A, Two major steps were proposed to explain the delivery of EV‐associated molecules to the nuclear compartment of recipient cells. First, the EVs are internalized by endocytosis at the plasma membrane (i). Second, once inside the endocytic pathway, a fraction of late endosomes (LE) penetrates the type II nuclear envelope invaginations where their content, notably the endocytosed EV‐associated molecules, are transferred into the nucleoplasm (ii). Two types of nuclear envelope invaginations are described. Type I invaginations (I) are those in which solely the inner nuclear membrane (INM) penetrates into the nucleoplasm, whereas type II invaginations (II) involve both the outer nuclear membrane (ONM) and INM. The endoplasmic reticulum (ER) is a continuation of ONM. B, Key players involved in the translocation of Rab7^+^ late endosomes to nuclear envelope invagination. Two proteins, vesicle‐associated membrane protein‐associated protein A (VAP‐A) and the cytoplasmic oxysterol‐binding protein‐related protein 3 (ORP3) forming a tripartite complex with late endosome‐associated Rab7 protein, are indispensable for the entry of late endosomes to the nuclear envelope invagination and/or their tether to ONM (I). Nuclear pores are somehow involved in the translocation of EV‐associated soluble (II) and membranous (III) cargo molecules into the nucleus. It remains to be explained how membranous components of EVs are extracted from the late endosomal membrane upon fusion of the former with the latter and the transport mechanism through nuclear pores, which are size restricted. C, Silencing CD9 in recipient cells and/or EVs or both interferes with the endocytosis of EVs and the nuclear transfer of their cargo molecules. Although the presence of divalent CD9 Ab stimulated these events with native cells and EVs, the lack of CD9 abrogated them.^23^ Panels A and B were modified from Ref.^29^

Our groups are studying EVs released by stem cells and cancerous cells, notably melanoma cells. We have extensively characterized those secreted by metastatic FEMX‐I cells. Electron microscopy examination has revealed the presence of a mixture of small and large EVs, suggesting that exosomes and ectosomes are simultaneously produced.^21^ The proteomic analysis of EVs, particularly those harbouring the stem (cancer stem) cell marker CD133,^22^ has defined their contents. They are particularly rich in tetraspanin proteins (CD9, CD63 and CD81) and in pro‐metastatic proteins, notably CD44, MAPK4K, ADAM10 and Annexin A2. Importin β1, a protein mediating nuclear transportation of cytoplasmic proteins through the nuclear pore complex, was also found therein. By monitoring the internalization of melanoma‐derived EVs and the intracellular routes of their content, particularly CD9 (see below), we discovered that EV‐associated proteins are transported into the nucleus of the host cell through late endosomes entering the nucleoplasmic reticulum (Figure 1A).^23^ Therein, EV‐associated cargo molecules can modify the gene expression of the host cells. These surprising findings are in line with numerous studies showing the atypical nuclear localization of the EV‐associated proteins CD9 and CD133 as well as the shuttling of proteins and nucleic acids to nucleoplasm of recipient cells.^3^, ^24^, ^25^, ^26^, ^27^, ^28^ Recently, we described that two proteins, ie vesicle‐associated membrane protein‐associated protein A (VAP‐A) and the cytoplasmic oxysterol‐binding protein‐related protein 3 (ORP3), are essential for the entry and the tethering of late endosomes to nuclear envelope invaginations of type II (Figure 1B). They form a tripartite complex with late endosome‐associated Rab7 proteins.^29^ Silencing VAP‐A or ORP3 abrogated the association of Rab7‐positive late endosomes with nuclear envelope invaginations, hence the transport of internalized EV‐derived cargo molecules to the nucleoplasm of recipient cells.^29^ The nuclear pores play a role in these processes given the treatment with importazole, a small molecule inhibitor of importin‐β‐mediated nuclear import, impaired the nuclear transfer of EV‐derived proteins.^23^ Finally, the initial internalization of CD9^+^ EVs occurs by endocytosis, which is an essential step for the nuclear localization of EV‐associated materials, given dynasore and methyl‐β‐cyclodextrin, two compounds known to inhibit the endocytosis mediated by clathrin/dynamin and lipid raft respectively, abrogated it.^23^

CD9 (alias Tetraspanin‐29, motility‐related protein‐1) is an integral membrane protein that is physiologically involved in cell fusion, adhesion and motility.^30^, ^31^, ^32^ For instance, CD9 has an important role in muscle cell fusion and in canine distemper virus and HIV‐1‐induced cell‐cell fusion.^33^, ^34^, ^35^ Depending on the context, CD9 functions have a metastasis suppressor or promoter activity (reviewed in Ref.^36^). CD9 has been extensively studied as a potential therapeutic target. Anti‐CD9 monoclonal antibodies (Ab) were found to specifically inhibit the trans‐endothelial migration of melanoma cells.^37^ We have shown that anti‐CD9, but not anti‐CD133, Ab enhances the nuclear uptake of EVs in recipient cells (Figure 1C).^23^ This effect is greater in melanoma cells than in mesenchymal stromal cells (MSCs), presumably because of the higher expression level of CD9 in cancer cells in comparison to stromal cells. Moreover, silencing CD9 in EVs and/or recipient cells strongly decreased the endocytosis of EVs and abolished the nuclear transfer of their contents, even in the presence of the anti‐CD9 Ab (Figure 1C).^23^

Here, we designed a strategy to block the uptake of EVs and the nuclear translocation of their cargos by recipient cells. To that aim, we generated an antigen‐binding fragment (Fab fragment; hereafter CD9 Fab) from 5H9 anti‐CD9 Ab (CD9 Ab), which could potentially saturate CD9 molecules present at the cell surface of host cells and EV‐associated ones and hence impair their function.^38^ The Ab and Fab fragment derived therefrom have been successfully employed for the treatment of different types of cancer, mainly through the inhibition of cell surface receptors.^39^ We report that monovalent CD9 Fab at doses achievable in vivo^40^ impedes the uptake of EVs in different melanoma cell lines and primary MSCs and consequently inhibits the nuclear transfer of their cargo proteins. Combined with other approaches, notably the direct targeting of cancer cells, such setting could lead to a new modality in cancer treatment by inhibiting the intercellular communication within the cancer cell niche.

## 2 METHODS

### 2.1 Cell culture

The FEMX‐I cell line was originally derived from the lymph node metastasis of a patient with malignant melanoma.^41^ FEMX‐I cells were highly metastatic in immunodeficient mice.^41^, ^42^ They were found to be wild‐type for BRAF, PTEN and NRAS.^23^, ^29^ The human A375 melanoma cell line was obtained from the American Type Culture Collection (catalog number #CRL‐1619^™^), whereas the human C8161 melanoma cell line was obtained from G. Pizzorno (University of Tennessee College of Medicine, Chattanooga, TN).^43^, ^44^ All cell lines were cultured in RPMI‐1640 (#10‐041‐CV; Corning Inc., Corning, NY) containing 10% foetal bovine serum (FBS; Atlanta Biologicals Inc., Flowery Branch, GA), 2 mmol/L l‐glutamine, 100 U/mL penicillin and 100 μg/mL streptomycin (Corning Inc.). Cells were used between passages 3 and 15. Cell lines were authenticated by morphology, proteomics and gene expression analysis as described.^45^ They were regularly tested for mycoplasma contamination using Venor^™^ GeM mycoplasma detection kit (Sigma‐Aldrich, St. Louis, MO).

Human bone marrow‐derived MSCs, isolated from bone marrow aspirates from normal adult donors after obtaining informed consent as described,^46^ were obtained from Dr. D. J. Prockop (Texas A&M) and prepared under a protocol approved by the Texas A&M Institutional Review Board. MSCs were used between passages 2 and 5. Their multipotency was regularly monitored by their differentiation into adipocytes and osteoblasts.^47^ MSCs and FEMX‐I cells expressing ectopically CD9‐green fluorescent protein (GFP) fusion protein were established as described.^23^ Under these conditions, almost all cells are positive. They were used to produce fluorescent EVs (see below). FEMX‐I cells depleted of CD9 by means of CD9 shRNA lentiviral particles were previously described.^23^ Approximately 85% of infected cells showed no CD9 expression (data not shown).

### 2.2 Production of CD9 antibody Fab fragment

Culture of 5H9 hybridoma cells^38^ and the production of CD9 Ab were performed at Mayo Clinic (Antibody Hybridoma Core, Rochester, MN). Conditioned media from hybridoma cultures growing in roller bottles in IMDM media (#12440‐053, Thermo Fisher Scientific, Gibco, Waltham, MA) containing 10% premium FBS (#S11150, Atlanta Biologicals Inc., Flowery Branch, GA) was pelleted in 250 mL centrifuge tubes at 1600 *g* The supernatant was clarified through 0.45‐μm Nalgene filters to remove remaining cell debris. The clarified supernatant was then passed through and bound to Protein G Sepharose FF HiLoad^™^ 26/40 columns (GE Healthcare, Pittsburgh, PA). Bound antibody was eluted with 100 mmol/L glycine buffer, pH 2.7. Eluted Ab was then immediately neutralized with 1 mol/L Tris‐HCl, pH 9 and desalted with HiPrep 26/10 columns (GE Healthcare). The buffer was exchanged with 1X PBS and the protein concentration was determined by measuring absorbance at 280 nm. Aliquots of the antibody (1 mg/mL) were stored at −80°C without addition of sodium azide.

The Fab fragment was generated using the Pierce Fab Purification kit (#44985; Thermo Fisher Scientific). Briefly, the CD9 Ab (500 μg) was incubated with papain immobilized on agarose resin for 3 hours at 37°C. The digested antibody was collected by centrifugation (5000 *g*, 1 minute) using a spin column and the flow through containing the antibody was placed in a new tube. The column was then washed once with PBS to recover any remaining antibody, which was pooled with the flow through. The fragment crystalline (Fc) fragment was then removed from digested antibody samples using NAb Protein A Plus Spin Column. After 10 minutes of centrifugation (1000 *g*), the Fab fragment found in the flow through was collected. The column was then washed twice with PBS. Each washing fraction was pooled with the Fab fraction. Antibody was concentrated using Microsep^™^ Advance Centrifugal Devices (10K molecular weight cut‐off; Pall Corporation). The final concentration of CD9 Fab was 0.75‐0.85 mg/mL. The Fab preparation was assessed using sodium dodecyl sulphate‐polyacrylamide gel electrophoresis (SDS‐PAGE) and Coomassie blue staining (see below).

### 2.3 SDS‐PAGE and immunoblotting

Preparation of Fab fragments was assessed using SDS‐PAGE under non‐reduced or reduced (ie in the presence of β‐mercaptoethanol) conditions. Samples were run on a 4%‐12% Bis‐Tris precast gel (Thermo Fisher Scientific, Life Technologies) and stained with Coomassie blue (Teknova, Hollister, CA) for 10 minutes. The gel was destained with 40% methanol/10% acetic acid solution.

**Figure 2:**
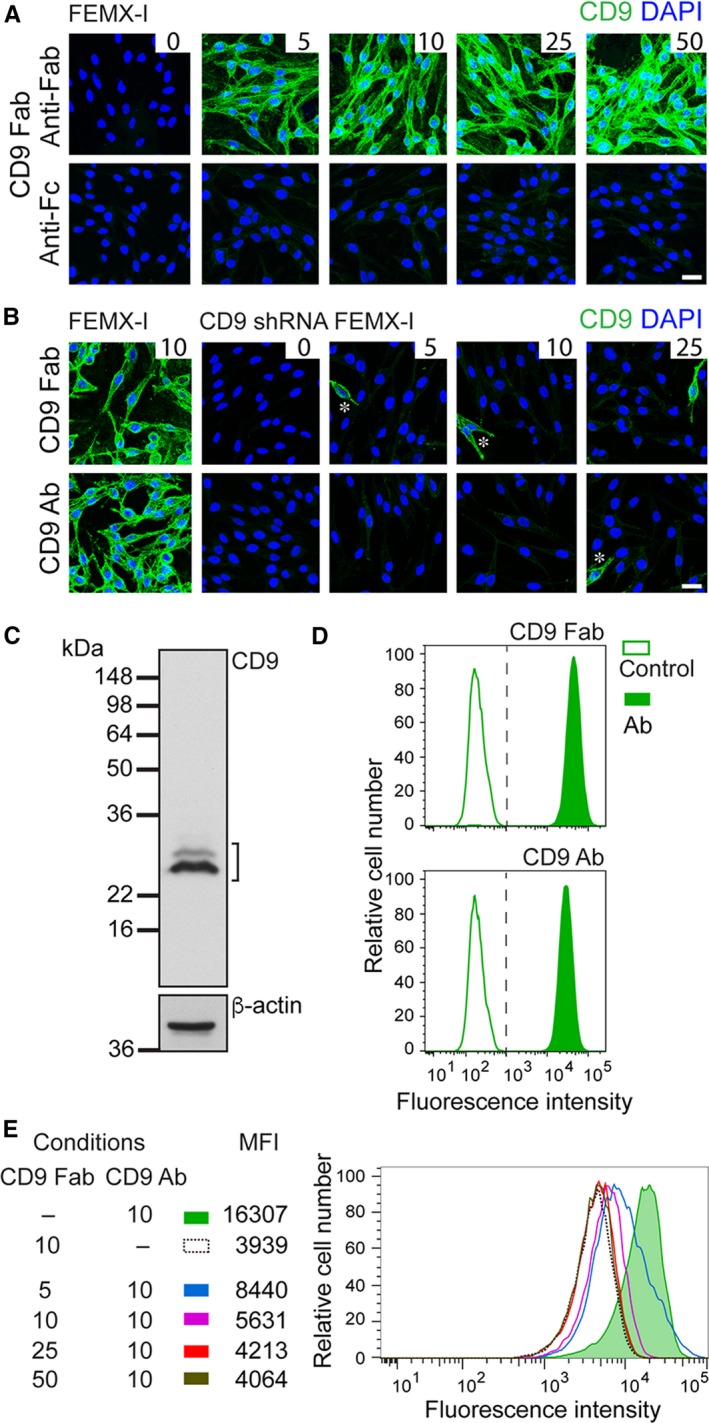
Characterization of CD9 Fab. A, Cell surface immunofluorescence on native FEMX‐I cells. FEMX‐I cells were surface labelled in the cold with CD9 Fab at different concentrations as indicated (μg/mL), PFA‐fixed and incubated with either anti‐Fab (top panels) or anti‐Fc (bottom panels) specific secondary conjugated to a fluorochrome (green). Nuclei were counterstained with 4′‐6‐diamidino‐2‐phenylindole (DAPI). B, Cell surface immunofluorescence on CD9‐depleted FEMX‐I cells. Native FEMX‐I cells and CD9 shRNA‐transduced cells were surface‐labelled in the cold with CD9 Fab (top panels) or CD9 Ab (bottom panels) at different concentrations (μg/mL), as indicated, PFA‐fixed and incubated with anti‐Fab or anti‐Fc specific secondary conjugated to a fluorochrome (green) respectively, prior to DAPI staining. Note that under these conditions, about 15% of infected cells still express CD9 in a proportion similar to native cells (asterisks). Scale bar, 25 μm. C, Immunoblotting. Detergent cell lysate (100‐μg protein) prepared from melanoma FEMX‐I cells was probed using Fab CD9 and horseradish peroxidase‐coupled anti‐Fab specific secondary antibody. β‐actin was used as control. Position of prestained molecular weight markers (kDa) are indicated. Bracket, CD9 immunoreactivity. D, Flow cytometry. FEMX‐I cells were surface labelled with either CD9 Fab (10 μg/mL, top) or CD9 Ab (10 μg/mL, bottom) followed by fluorochrome‐conjugated anti‐Fab or anti‐Fc specific secondary antibody respectively. E, CD9 Fab inhibits the cell binding of native CD9 Ab. FEMX‐I cells were sequentially labelled with CD9 Fab at different concentrations as indicated (μg/mL) followed by CD9 Ab (10 μg/mL) and fluorochrome‐conjugated anti‐Fc specific secondary antibody. Samples were analysed using flow cytometry. The median fluorescence intensity (MFI) is indicated. As negative and background controls, primary Ab (D) or CD9 Ab (E) was omitted

Cells were solubilized in lysis buffer (1% Triton X‐100, 100 mmol/L NaCl, 50 mmol/L Tris‐HCl, pH 7.5) supplemented with the Set III protease inhibitor cocktail (Calbiochem, Burlington, MA) for 30 minutes on ice. Cell lysates were centrifuged at 12 000 *g* for 10 minutes in 4°C. The supernatant was collected and Laemmli sample buffer without reducing agent was added. Proteins were separated using either 12% SDS‐PAGE gel (Figure 2 and Figure S1) or a precast gel (see above; Figure S3) along with the Trident prestained protein molecular weight ladder (GeneTex, Irvine, CA) and transferred overnight at 4°C to a nitrocellulose membrane (Thermo Fisher Scientific) or poly(vinylidene difluoride) membrane (Millipore, Bedford, MA: pore size 0.45 μm). After transfer, membranes were incubated in a blocking buffer (PBS containing 1% bovine serum albumin [BSA] or 5% low fat milk powder and 0.3% Tween 20) for 60 minutes at room temperature (RT). Afterward, the membranes were probed using either primary CD9 Fab (1 μg/mL) generated from mouse 5H9 Ab (see above) or commercial mouse anti‐CD9 (clone P1/33/2, #sc‐20048; Santa Cruz Biotechnology, Santa Cruz, CA) or anti‐β‐actin (clone C4, #sc‐47778; Santa Cruz Biotechnology) Ab for 60 minutes at RT. After three washing steps of 10 minutes each with PBS containing 0.1% Tween 20, the antigen‐antibody complexes were detected using two protocols. In the case of CD9 Fab, we used goat anti‐mouse Fab specific horseradish peroxidase (HRP)‐conjugated secondary antibody (#A2304; Sigma‐Aldrich), which was visualized with enhanced chemiluminescence reagents (ECL system; Amersham Corp., Arlington Heights, IL). The membranes were exposed to films (Hyperfilm ECL; Amersham‐Pharmacia). With other Abs, the IRDye 680RD anti‐mouse IgG (#926‐68070; LI‐COR Biosciences, Lincoln, NE) was applied. Membranes were washed thrice (10 minutes each) in PBS containing 0.1% Tween 20, rinsed in ddH_2_O and antigen‐antibody complexes were visualized using an Odyssey CLx system (LI‐COR).

### 2.4 Production of CD9‐GFP^+^ EVs

Extracellular vesicles were enriched by differential centrifugation from 72 hour‐conditioned media (serum‐free Dulbecco's modified eagle medium [DMEM]/Ham's F‐12 1:1, supplemented with 2% B‐27 [Thermo Fisher Scientific]) of engineered FEMX‐I cells and MSCs expressing CD9‐GFP as described previously.^21^, ^22^ Conditioned medium was centrifuged at 10 000 *g* for 30 minutes at 4°C and the resulting supernatant was centrifuged at 200 000 *g* for 60 minutes at 4°C. The pellet was re‐suspended in 200 μL of PBS. To determine the EV concentration, we used the light‐scattering characteristics of 488‐nm laser light on EV preparations undergoing Brownian motion injected by continuous flow into the sample chamber of a Nanosight LM10 unit (Malvern Panalytical Inc., Westborough, MA). The calculated EV concentration was an average of six 30‐second video recordings. As described previously, the average size of EVs produced by FEMX‐I cells and MSCs was 123 and 114 nm respectively.^23^ Those produced by FEMX‐I cells were formerly characterized by electron microscopy.^21^

### 2.5 Incubation of cells with EVs

Cells (1 × 10^5^) were plated into 35‐mm microscopy dishes containing 0.17‐mm thick glass coverslips on the bottom and incubated overnight at 37°C to allow complete cell adherence (MatTek Corporation, Ashland, MA). Afterward, they were incubated with various concentrations of CD9‐GFP^+^ EVs (eg, 5 × 10^7^ particles per mL [0.075 μg protein per mL]; 2.5 × 10^8^ particles per mL [0.375 μg protein per mL] or 1 × 10^9^ particles per mL [1.5 μg protein per mL]) for 5 hours at 37°C prior to fixation. EVs were derived from the same cell type as used for the recipients except for A375 and C8161 cells in which EVs were produced from CD9‐GFP transfected FEMX‐I cells. In some experiments, EVs and/or cells were pre‐incubated with CD9 Fab or CD9 Ab at various concentrations as indicated for 30 minutes at 4 and 37°C respectively. The EVs and cells were then incubated together in the presence of antibodies (or without as control) for 5 hours at 37°C prior to analysis.

### 2.6 Confocal laser scanning microscopy and fluorescence quantification

Cell surface immunolabelling of native or CD9‐depleted FEMX‐I cells was performed as described.^48^ Briefly, cells growing on fibronectin‐coated coverslips were washed with ice‐cold PBS containing 1 mmol/L CaCl_2_ and 0.5 mmol/L MgCl_2_ (Ca/Mg‐PBS) and incubated in blocking buffer I (Ca/Mg‐PBS containing 0.2% gelatin) for 10 minutes. Cells were then incubated for 30 minutes with CD9 Fab or CD9 Ab at different concentrations (eg, 5, 10, 25 and 50 μg/mL) diluted in blocking buffer. The whole procedure was performed at 4°C. Afterward, they were fixed in 4% paraformaldehyde (PFA) for 30 minutes at RT, quenched with 50 mmol/L NH_4_Cl for 10 minutes, washed in PBS and incubated in blocking buffer II (PBS containing 0.2% gelatin) for 20 minutes. Samples were incubated with fluorescein isothiocyanate (FITC)‐conjugated secondary antibody specific either for the mouse Fab or Fc fragment (#F4018, #F5387 respectively, 1:200; Sigma‐Aldrich) diluted in blocking buffer II. Nuclei were labelled with 4′‐6‐diamidino‐2‐phenylindole (1 μg/mL; Sigma‐Aldrich). Cells were washed with PBS and distilled water then mounted in Mowiol 4.88 (Merck, Darmstadt, Germany). Images were captured with Leica SP5 upright confocal microscope under the same settings for both Fab‐ and Fc‐specific secondary antibody labelling. Composites of 27‐30 optical sections are shown (Figure 2A,B). The images were prepared using Fiji^49^ and Adobe Illustrator software.

Alternatively, cells incubated with CD9‐GFP^+^ EVs (see above) were fixed in 4% PFA and afterward permeabilized with 0.2% Tween 20 diluted in PBS (permeabilization buffer). Both steps were performed for 15 minutes at RT. They were then incubated in blocking buffer III (PBS containing 1% BSA) and labelled with mouse anti‐SUN2 Ab (clone A‐10, #sc‐515330; Santa Cruz Biotechnology) for 60 minutes each step at RT. Cells were washed twice with PBS, incubated with tetramethylrhodamine (TRITC)‐conjugated anti‐mouse IgG (#715‐025‐150; Jackson ImmunoResearch, West Grove, PA) or Cy5‐conjugated anti‐mouse IgG (#715‐175‐150; Jackson ImmunoResearch) secondary antibodies for 30 minutes and again washed twice prior to observation. All antibodies were diluted in permeabilization buffer containing 1% BSA. Cells were imaged in PBS using confocal laser scanning microscopy (CLSM) using a Nikon A1R+ inverted confocal microscope with a 60X Apo‐TIRF oil‐immersion objective and a numerical aperture of 1.49 at either 512 × 512 or 1024 × 1024 pixel resolution. Solid‐state lasers of 488, 561 and 638 nm solid‐state lasers were used to excite GFP, TRITC and Cy5 respectively and corresponding fluorescence emissions were collected using 500‐550, 570‐620 and 662‐737 nm long pass filters.

All images were acquired under the same microscope settings for subsequent calculations of mean fluorescence intensity and recorded using NIS Elements software (Nikon). Raw images were processed using Fiji. Each optical section through the cell (21 sections of 0.4 μm for cancer cells and 0.2 μm for MSCs) was assessed individually. Any observed GFP fluorescent signal was counted as EV‐derived biomaterials and data collectively calculated. To count nuclear fluorescent materials, a region of interest (ROI) was drawn along the nucleus on each optical section and an auto threshold generated by Fiji was applied. Positive signals were counted using the “analyze particle” function. To determine the value of cytoplasmic GFP fluorescence for each cell, an ROI was also drawn around the cytoplasm, using the cell border as a guide, but excluding the nucleus.

### 2.7 Flow cytometry

FEMX‐I cells were trypsinized using 0.05% trypsin with 0.53 mmol/L EDTA (Corning Inc.), washed twice in PBS and re‐suspended in PBS containing 1% BSA. Cell suspension aliquots of 100 μL (1 × 10^6^ cells) were incubated with either CD9 Fab or CD9 Ab (clone 5H9) (both at 10 μg/mL in PBS containing 1% BSA) for 30 minutes at 4°C. After two washing steps with PBS, samples were incubated with FITC‐conjugated secondary antibody specific either for the mouse Fab or Fc fragment (see above, 1:600) for another 30 minutes at 4°C. As negative controls, primary Ab was omitted. For competitive inhibition experiment, cells were incubated first with CD9 Fab at different concentrations (0, 5, 10, 25 and 50 μg/mL) and then with CD9 Ab (10 μg/mL) followed by Fc‐specific FITC‐conjugated secondary antibodies. All incubations were performed for 30 minutes at 4°C. To set up the background staining reminiscent of a residual undigested CD9 Ab in CD9 Fab preparation, we omitted CD9 Ab. After washing with PBS, 20 000 events were acquired on a LSRII flow cytometer (BD Biosciences). Instrument settings and gating strategies were established using cells incubated solely with individual secondary antibody as negative controls. Data were analysed using FlowJo software (TreeStar, Ashland, USA). Median fluorescence intensity (MFI) was calculated as a difference of MFI values of stained and negative control populations.

To determine the amount of cell surface CD9 molecules in a given cell, Quantum^™^ Simply Cellular^®^ anti‐mouse IgG kit (#815; Bangs Laboratories Inc., Fishers, IN) was utilized. Cells (1 × 10^5^) and 4 microsphere populations, containing increasing levels of Fc‐specific capture antibody, were incubated with phycoerythrin‐conjugated anti‐CD9 Ab (clone M‐L13, #555372; BD Biosciences, San Jose, CA) in PBS containing 0.5% BSA for 30 minutes on ice. Both cells and microspheres were then analysed using flow cytometry using the same settings as above according to manufacturer's instructions. A standard curve was generated using the median channel values of the microspheres and the amount of CD9 molecules per cell was determined from this curve. All calculations were performed with the QuickCal analysis program provided in the kit.

### 2.8 Statistical analysis

All experiments were performed at least in triplicate. A minimum of 30 cells was analysed in each experiment. Error bars in graphical data represent means ± standard deviation. Statistical analysis was determined by one‐way analysis of variance followed by pairwise comparison of means with Dunnett's multiple comparison adjustment using the statistical program Stata 12 (StataCorp LLC, College Station, TX). *P*‐values inferior to 0.05 were considered significant.

## 3 RESULTS

### 3.1 Generation of CD9 antibody Fab fragment

Given the positive impact of divalent Ab directed against CD9 on the uptake of CD9^+^ EVs by melanoma cells and the negative impact upon silencing CD9 on either EVs or recipient cells,^23^ we sought whether CD9 Fab could influence the internalization and consequently the intercellular transfer of EV‐associated cargo molecules. To investigate this issue, we generated CD9 Fab from 5H9 Ab, which recognizes an unidentified epitope in the extracellular part of CD9 (Figure S1A).^38^ The Ab (IgG_1_ kappa) produced from hybridoma clone 5H9 was digested with papain to generate the Fab and Fc fragments. The latter were removed selectively using immobilized protein A (Figure S1B). As observed using SDS‐PAGE under non‐reducing and reducing conditions, the 5H9 Ab was successfully digested and the 50‐kDa CD9 Fab was isolated (Figure S1C).

### 3.2 Characterization of CD9 Fab

To determine the functionality of CD9 Fab, we evaluated its binding to melanoma FEMX‐I cells by various methods. First, cells growing on fibronectin‐coated support were surface labelled in the cold with CD9 Fab at different concentrations followed by a fluorochrome‐conjugated secondary antibody specific either for the mouse Fab or Fc fragment. The CLSM analysis revealed that the antigen‐CD9 Fab complex is recognized by anti‐Fab secondary antibody already at low concentration of primary Ab (Figure 2A, top panels). In contrast, solely a very weak labelling was detected with a secondary antibody directed against mouse Fc, indicating the effective papain digestion of CD9 Ab (Figure 2A, bottom panels). When a similar experiment was performed with CD9‐depleted FEMX‐I cells,^23^ almost no immunolabelling was detected either with CD9 Fab or full‐length antibody (Figure 2B, top and bottom panels respectively). Second, we analysed whether CD9 Fab can recognize CD9 by immunoblotting. To that end, detergent lysate prepared from FEMX‐I cells was resolved on SDS‐PAGE under non‐denaturing condition and probed with CD9 Fab. As shown in Figure 2C, CD9 Fab recognized the CD9 molecules. Third, we evaluated the capacity of CD9 Fab to detect its antigen using flow cytometry. A suspension of FEMX‐I cells was subjected to immunolabelling in the cold using either CD9 Fab or full CD9 Ab followed by fluorochrome‐conjugated secondary antibody specific either for mouse Fab or Fc fragment respectively. As negative control, primary antibody was omitted. Flow cytometry analyses indicated that CD9 Fab could detect CD9^+^ cells similar to the native anti‐CD9 Ab (Figure 2D). Altogether, these experiments demonstrated that CD9 Fab recognizes its antigen under various conditions, notably its native conformation.

### 3.3 CD9 Fab interferes with the cell binding of native CD9 antibody

Can CD9 Fab interfere with the binding of corresponding native CD9 Ab? To address this issue, we pre‐incubated FEMX‐I cells in suspension with various concentrations of CD9 Fab prior to the addition of CD9 Ab and fluorochrome‐conjugated secondary antibody specific for the Fc fragment. Samples were analysed by flow cytometry. As a positive control, CD9 Fab was omitted whereas CD9 Ab was absent for the background control. As shown in Figure 2E, CD9 Fab blocked the binding of the native Ab in a dose‐dependent fashion, indicating that it could specifically label the cell surface CD9 molecules. We concluded that the monovalent CD9 Fab could be useful in achieving our objective, ie interfering with the uptake of CD9^+^ EVs.

### 3.4 Differential effect of CD9 Fab versus native antibody on the internalization of EVs

**Figure 3:**
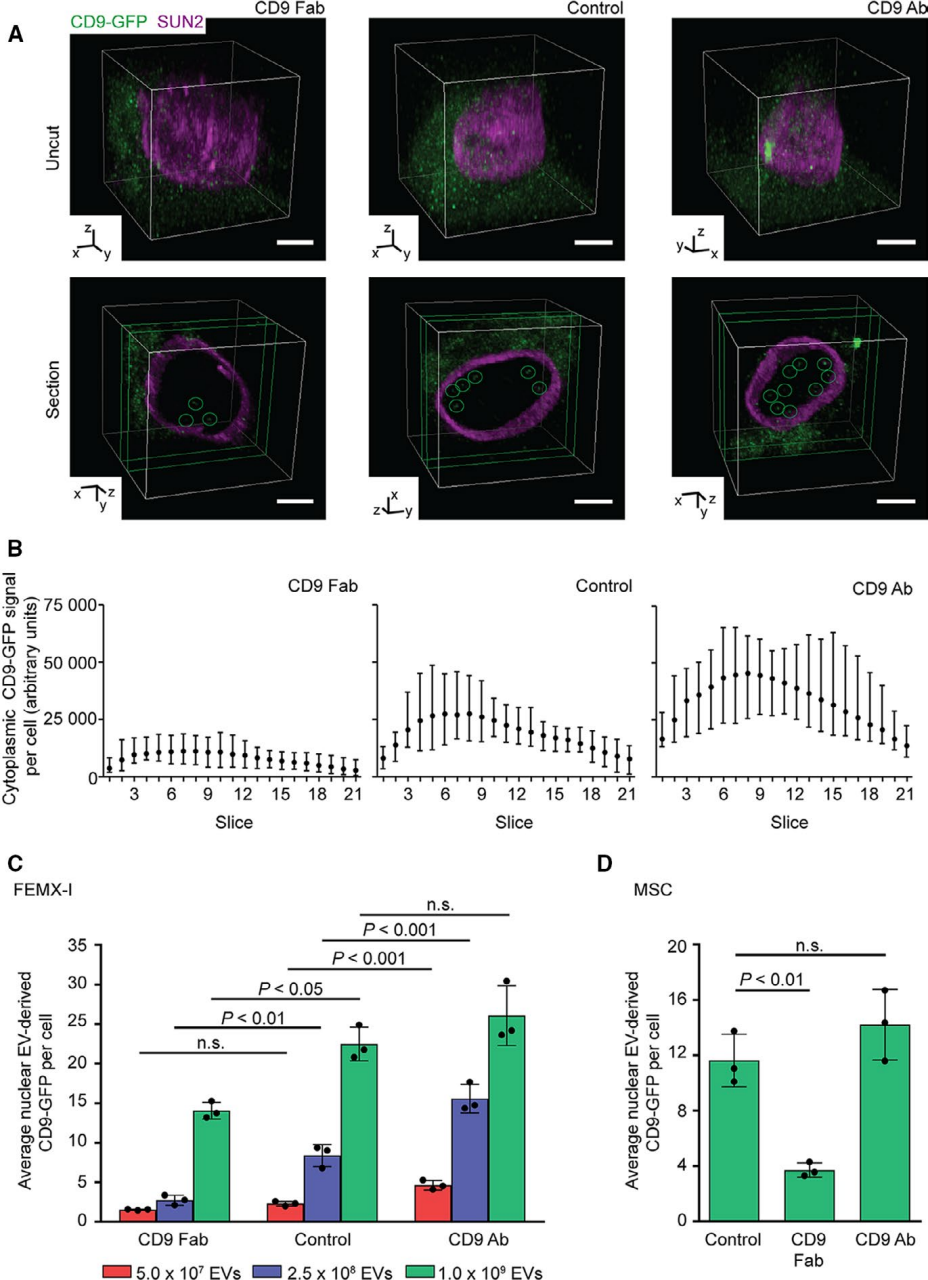
CD9 Fab impedes the uptake of extracellular vesicles (EVs) and nuclear transfer of their cargo proteins in melanoma and stromal cells. A‐C, FEMX‐I cells were pre‐incubated (30 min) without (control) or with CD9 Fab or CD9 Ab (25 μg/mL) prior to the exposure to fluorescent EVs derived from CD9‐GFP ^+^ FEMX‐I cells for 5 h. Different concentrations of EVs were used (A‐C, 2.5 × 10^8^ particle per mL [blue]; C, 5.0 × 10^7^ [red] or 1.0 × 10^9^ particle per mL [green]). Samples were then fixed and immunolabelled for SUN2 prior to confocal laser scanning microscopy. A three‐dimensional reconstruction of the entire cell (uncut) or three sections (0.4‐μm slices each, section) is shown (A). CD9‐GFP appears as discrete punctate signals either in the cytoplasm or nucleoplasm (circles) of recipient cells. The amount of CD9‐GFP signal was quantified using serial optical sections through a cell using the cytoplasmic (B) and nuclear (C) compartments as a region of interest (see Figure S2B). Mean with the range of fluorescence per slice from 10 individual cells are shown (B). D, Native MSCs were exposed to EVs (1.0 × 10^9^ particle per mL) derived from CD9‐GFP ^+^ MSCs upon their pre‐incubation without or with CD9 Fab or CD9 Ab as described above. Punctate nuclear CD9‐GFP signal per cell was quantified. Means ± SD are shown (C, D). 50 (C) or 20 (D) cells were evaluated per experiment (n = 3). *P‐*values are indicated. N.S., not significant. Scale bars, 5 μm

**Figure 4:**
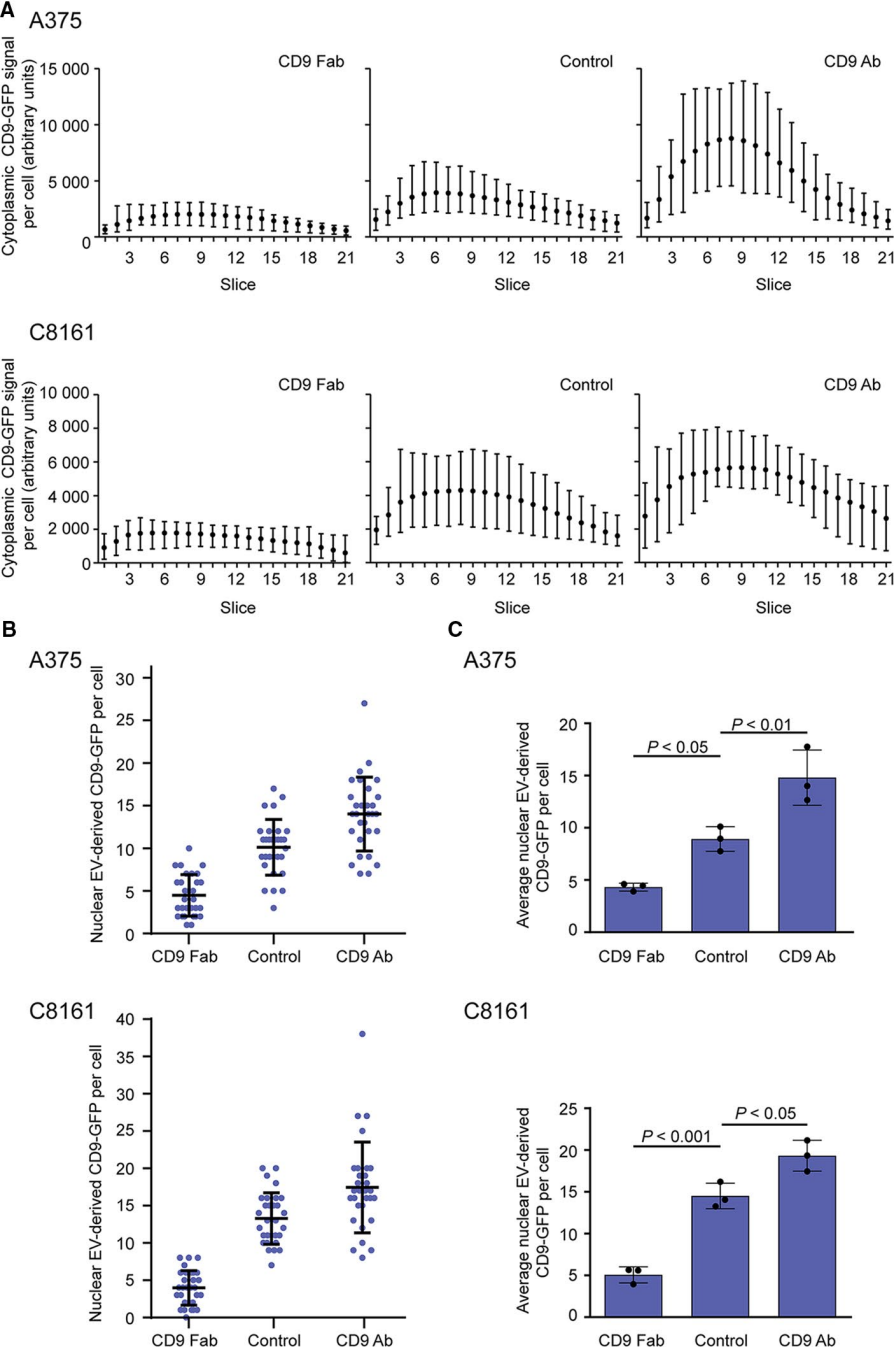
CD9 Fab impedes the uptake of extracellular vesicles (EVs) and nuclear transfer of their cargo proteins in various malignant melanoma cells. (A‐C) Melanoma A375 or C8161 cells were incubated (30 min) without (control) or with CD9 Fab or CD9 Ab (25 μg/mL) prior to the exposure to fluorescent EVs (2.5 × 10^8^ particle per mL) derived from FEMX‐I cells expressing CD9‐GFP for 5 h. Samples were then fixed and immunolabelled for SUN2 prior to confocal laser scanning microscopy. Cytoplasmic (A) and nuclear (B, C) CD9‐GFP signals per cell were quantified using Fiji. Means with the range of fluorescence per slice from 10 individual and representative cells are shown (A). 30 cells were evaluated per condition and experiment (B) and the means ± SD of three independent experiments are shown (C). *P‐*values are indicated

To determine the impact of CD9 Fab on the internalization of EVs by melanoma cells, we used engineered FEMX‐I cells to express the CD9‐GFP fusion protein.^23^ These cells release in vivo‐labelled fluorescent EVs that could be used to monitor EV uptake upon incubation with recipient cells. CD9‐GFP^+^ EVs released in the conditioned culture media were enriched by differential centrifugation (for details see Methods,^23^). Prior to the exposure of native FEMX‐I cells to CD9‐GFP^+^ EVs, cells were pre‐incubated for 30 minutes at 37°C with either CD9 Fab or CD9 Ab (25 μg/mL). As control, no antibody was added. Afterward, cells were incubated with CD9‐GFP^+^ EVs (2.5 × 10^8^ particle per mL) without removing the antibodies for 5 hours and then fixed, immunolabelled for protein SUN domain‐containing protein 2 (SUN2), an inner nuclear membrane protein and analysed using CLSM. At first glance, we noticed that the uptake of CD9‐GFP^+^ EVs by recipient cells seemed variable under the native conditions, ie without the addition of CD9 Ab. Therein, GFP fluorescence appears as strong, medium or weak among cells (Figure S2A). In contrast, GFP fluorescence becomes more homogeneous within the cell population upon the addition of antibodies. A three‐dimensional reconstruction of labelled recipient cell revealed that CD9‐GFP signal associated with their cytoplasm was considerably reduced in the presence of CD9 Fab by comparison to control (Figure 3A, uncut)—for an overview see Figure S2A. Quantification of each optical section confirmed it (Figure 3B). In contrast, the presence of CD9 Ab yielded the opposite effect, ie, an increase of cytoplasmic CD9‐GFP was detected (Figure 3A, uncut; 3B). Interestingly, similar outcome were observed with two other melanoma cell lines, A375 and C8161, exposed to FEMX‐I cell‐derived CD9‐GFP^+^ EVs (Figure 4A), indicating that CD9 Fab inhibits the uptake of EVs.

### 3.5 CD9 Fab inhibits the nuclear transfer of EV‐derived cargo proteins

We previously reported that cargo proteins derived from EVs are not only internalized by host cells, but also a fraction of them is transferred to their nucleoplasm by the intermediate of late endosomes entering into nucleoplasmic reticulum.^23^, ^29^ Does CD9 Fab interfere with this mechanism? The analysis of the nuclear compartment of melanoma cells pre‐treated with monovalent or divalent Abs prior to incubation with CD9‐GFP^+^ EVs (2.5 × 10^8^ particle per mL) showed a decrease or an increase in the CD9‐GFP^+^ signals in the nucleoplasm respectively, compared to the control (Figure 3A, section, green circle; 3C; see also Table 1). As previously demonstrated,^23^, ^29^ CD9‐GFP^+^ signal in the nuclear compartment appeared with a punctate pattern (Figure 3A; Figure S2B, green circle).

**Table 1:** Differential impact of CD9 antibody on the nuclear localization of extracellular vesicles (EV)‐derived cargo protein

Antibody	Experimental procedure^a^	Average nuclear EV‐derived CD9‐GFP per cell^c^	*P‐*values (relative to control)	*P‐*values (relative to procedure A)
Control^b^	A	8.42 ± 0.74	—	
	B	8.41 ± 0.61	—	
	C	8.02 ± 0.44	—	
CD9 Fab	A	2.89 ± 0.13	0.05	
	B	2.99 ± 0.45	0.001	
	C	1.53 ± 0.09	0.0001	0.05
CD9 Ab	A	15.67 ± 1.20	0.005	
	B	14.17 ± 0.76	0.001	
	C	14.20 ± 0.32	0.0001	0.5

a A, Cells were pre‐incubated with antibody (25 μg/mL, 30 min, 37**°**C) before the addition of CD9‐GFP^+^EVs (5 h). B, CD9‐GFP^+^EVs were pre‐incubated with antibody (25 μg/mL, 30 min, 4**°**C) before their incubation with cells (5 h). C, Cells and CD9‐GFP^+^EVs were pre‐incubated with antibody (12.5 μg/mL each, 30 min, 37 or 4**°**C respectively) before their co‐culture (5 h).

b Control refers to the three experimental procedures (A‐C) without the addition of antibody.

c At least 30 cells were evaluated per condition (n = 3).

The addition of different amounts of CD9‐GFP^+^ EVs (eg, 5.0 × 10^7^ or 1.0 × 10^9^ particle per mL) was also evaluated in FEMX‐I cells. In most cases, the numbers of nuclear CD9‐GFP were significantly lower or higher in cells exposed to CD9 Fab or CD9 Ab respectively (Figure 3C). Only with a high amount of EVs (ie 1.0 × 10^9^ particle per mL) no significant difference was observed between CD9 Ab and control. Similar observations were made with A375 and C8161 cells (Figure 4B,C).

When the same experiments were performed with primary MSCs as recipient cells as well as donor cells for fluorescent EVs (1 × 10^9^ particle per mL), we observed also a significant decrease in nuclear and cytoplasmic CD9‐GFP in cells pre‐treated with CD9 Fab (25 μg/mL) (Figure 3D; data not shown). The CD9 Ab did not significantly increase the EV uptake which can be explained by a limited quantity of CD9 molecules in MSCs in comparison to melanoma cells, as observed by immunoblotting and quantitative fluorescence analyses using flow cytometry (Figure S3A‐C).

### 3.6 A minimal concentration of CD9 Fab is necessary to interfere with EV uptake

**Figure 5:**
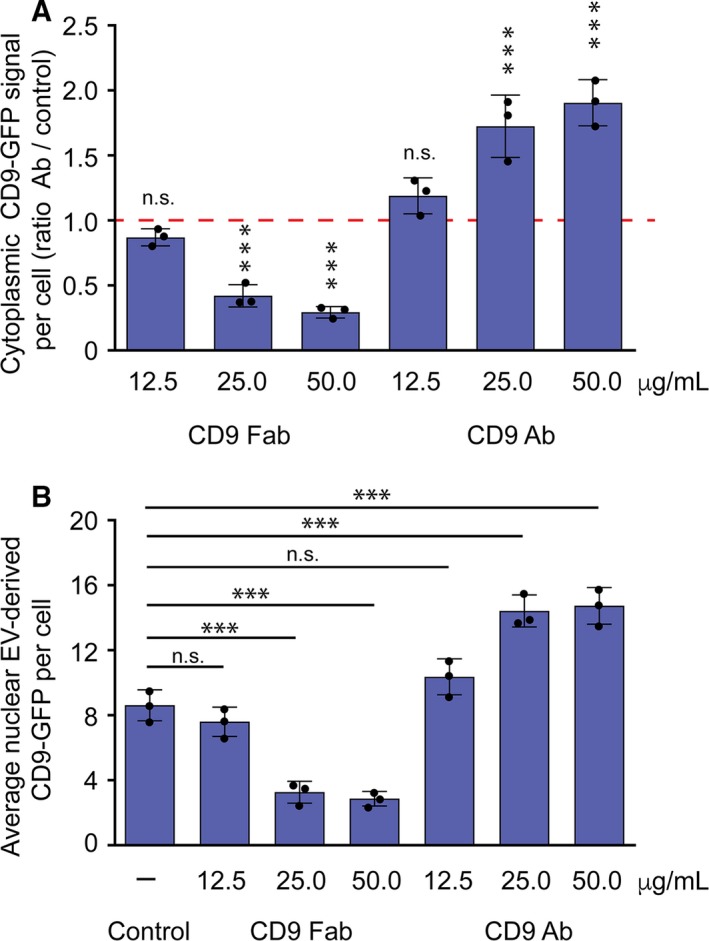
Dose‐dependent inhibition of CD9 Fab on the extracellular vesicles (EV) uptake and nuclear transfer of their cargo proteins. (A, B) FEMX‐I cells were pre‐incubated (30 min) with different concentration of CD9 Fab or CD9 Ab as indicated prior to the exposure to CD9‐GFP ^+^ EVs (2.5 × 10^8^ particles per mL) for 5 h. As control, no antibody was added (–). Cytoplasmic (A) and nuclear (B) CD9‐GFP signals per cell were quantified using Fiji. Means ± SD are shown. 10 (A) or 30 (B) cells were evaluated per experiment (n = 3). They were evaluated in comparison to the control (A, red line; B, –). ***, *P *≤* *0.001. N.S., not significant

We assessed whether the uptake of EVs is dependent on the concentration of CD9 Fab. FEMX‐I cells were subjected to increasing concentrations of CD9 Fab or CD9 Ab prior to incubation with CD9‐GFP^+^ EVs (2.5 × 10^8^ particle per mL). As shown in Figure 5A, the uptake of EVs was progressively inhibited as the concentration of CD9 Fab increased, whereas the opposite effect was again observed in cells treated with CD9 Ab, ie more EVs were internalized with increasing CD9 Ab concentration. A similar trend was observed in the number of CD9‐GFP signals in the nuclear compartment (Figure 5B). These results are in line with the interference of CD9 Fab to cell surface CD9 Ab binding observed using flow cytometry (Figure 2E). Thus, a minimal amount of antibody (ie 25 μg/mL) seems to be indispensable to inhibit (or promote) the EV uptake.

Lastly, we determined whether the pre‐incubation of EVs with Ab (25 μg/mL) or of both EVs and cells individually, instead of cells only as performed until now, influenced internalization and the nuclear localization of EV‐derived cargo proteins. We wanted to rule out a potential negative impact of the addition of the Abs (CD9 Fab or CD9 Ab) to recipient cells, which could stimulate the internalization of cell surface CD9, hence limit the EV uptake. If it turned out to be the case, all acquired numbers would be underestimated. Similarly, we wished to exclude that the addition of Abs, particularly CD9 Ab, to EVs would reduce their internalization by favouring, for instance, their clustering. As presented in Table 1, we found that the pre‐incubation of cells with Abs did not influence the final outcome when compared to the pre‐incubation of EVs (procedure A vs. B). However, the nuclear localization of EV‐derived cargo proteins was significantly reduced when both entities (EVs and cells) were pre‐incubated individually with the monovalent, but not the divalent, Ab (see procedure C by comparison to A).

## 4 DISCUSSION

**Figure 6:**
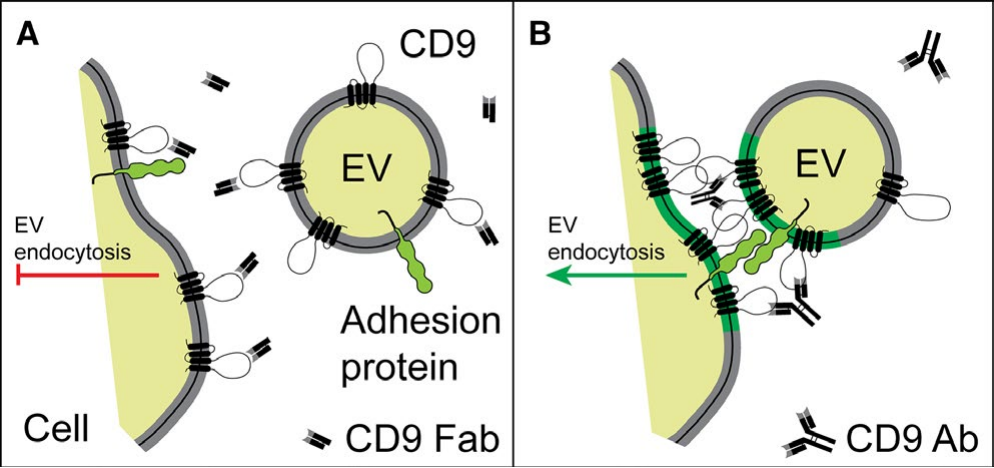
Schematic representation showing the negative and positive impact of CD9 Fab and CD9 Ab, respectively, on the endocytosis of CD9‐containing extracellular vesicles (EVs). (A, B) CD9 Fab will saturate CD9 proteins present at the surface of cells and EVs, hence interfere with its CD9 function. CD9 Fab can impede the cis/trans‐dimerization of CD9, its oligomerization and/or its interaction with other protein partners and block EV endocytosis (red bar). In contrast, divalent CD9 Ab could cross‐link CD9 proteins associated with host cells and EVs and consequently stimulate the endocytosis on EVs (green arrow). In the latter case, cis‐dimerization/oligomerization of CD9 might organize the components (proteins and lipids) of plasma membrane and/or EV membrane into a specific tetraspanin web (green segment), whose constituents, notably potential adhesion proteins as illustrated would somehow regulate the cell‐EV interaction and promote the endocytosis of EVs

In this study, we demonstrated that a monovalent Ab directed against tetraspanin CD9 interferes with the uptake of EVs by cancer cells and primary MSCs as well as with the nuclear transfer of their cargo proteins. The latter event is probably a direct consequence of the endocytosis inhibition of EVs.^23^ Under these conditions, CD9 Fab could saturate the CD9 molecules located at the surface of cells and EVs and consequently interfere negatively with its function (Figure 6A; see below). The synergic impact of the pre‐incubation of cells and EVs individually with CD9 Fab is consistent with this scenario. Our data are in line with an elegant study showing the CD9 Fab can inhibit the transfer of materials between CD9‐containing membranous vesicles, called epididymosomes and maturing epididymal spermatozoa.^50^ In contrast, divalent CD9 Ab promotes these events, which can be correlated to antibody‐induced cross‐linking of CD9 associated with EVs and host cells (Figure 6B). Does CD9 play a role in the initial adhesion of EVs to the recipient cell? The earlier observation made with sperm‐egg fusion suggests it. Jégou and colleagues demonstrated that the fertilization process is controlled by sperm‐egg adhesion properties driven by CD9.^51^ In such process, CD9 might organize the components (proteins and lipids) of plasma membrane and/or EV membrane into a specific tetraspanin web (Figure 6B, green), whose constituents (eg, adhesion proteins) would somehow regulate the interaction with EVs and promote their endocytosis.^52^, ^53^, ^54^, ^55^ Similarly, CD9 has been proposed to act as a scaffold in the regulation of adhesion molecules at the immune synapse and T lymphocyte activation.^56^ It remains to be determined whether the cis‐dimerization of CD9 in the membrane of recipient cells as well as in EVs is involved.^57^ We could not exclude that a trans‐dimerization of CD9, ie molecules expressed in opposite membranes, occurs. Indeed, our present data with divalent Ab as mentioned above and the complete lack of EV endocytosis previously observed in melanoma cells in which CD9 was silenced in both entities (cells and/or EVs), suggest it (Figure 1C).^23^ It will be of interest to investigate the CD9 cis/trans‐dimerization by co‐immunoprecipitation using engineered CD9 proteins associated with cells and EVs with distinct epitope tags.

Besides the exact molecular mechanism regulating the adhesion of EVs to recipient cells and their internalization, it will be of interest to determine whether other anti‐CD9 antibodies interfere with the EV uptake and nuclear transfer of their cargo proteins, as observed here with CD9 Fab derived from 5H9 Ab. The proper localization of their respective epitope might be crucial to promote these effects and it is conceivable that distinct CD9 Fab (or again other CD9 interacting partners) could potentially synergize their inhibitory effect. Other tetraspanin proteins enriched in EVs such as CD81 should also be evaluated in this respect.

The intercellular transfer of materials by cancer cell‐produced EVs played a significant role in the transformation of microenvironment, notably in the bone marrow, to favour metastasis and tumour growth.^7^ Interfering locally with these mechanisms, particularly the internalization of cancer cell‐derived EVs by MSCs, one of the main targeted cellular constituents of tumour niche,^58^ could find a cutting‐edge clinical application. MSCs have an important role in co‐ordinating the tumour microenvironment. Transformed MSCs produced growth factors favouring tumour growth and angiogenesis, inhibited anti‐tumour immune responses and shaped the tumour inflammatory environment.^59^, ^60^, ^61^ Thus, our data with MSCs exposed to CD9 Fab might find new avenues to prevent the bone marrow transformation. In addition to cancers, other diseases involving the intercellular transfer of biomaterials mediated by EVs, such as neurodegenerative diseases (eg, Parkinson's disease, Alzheimer's disease, amyotrophic lateral sclerosis), could benefit from this new potential therapeutic approach.^62^, ^63^

Finally, our observations could benefit the areas of regenerative medicine and tissue engineering. Here, the stimulation of EV endocytosis by specific divalent antibodies could favour tissue/organ repair.^64^ Myocardial regeneration might be a good example for such intervention with MSCs as a promising source of donor cell EVs.^65^, ^66^ Such approach could be an interesting alternative to stem cell‐based therapy.

## ACKNOWLEDGEMENTS

The authors wish to thank Fabio Anzanello for skillful technical assistance, Emmett Findlay and Wolfgang Gilliar for their constant support and Thomas G. Beito of the Mayo Antibody Hybridoma Core for preparation and purification of the anti‐CD9 Ab from the 5H9 hybridoma.

## CONFLICTS OF INTEREST

United Kingdom patent application number GB1814065.7 and United States provisional patent number 62/724 183 are pending. We have no other potential conflict of interest.

## AUTHOR CONTRIBUTIONS

MFS, GR, DC, AL conception and design; MFS, JK, DC, AL development of methodology; MFS, JK, CM acquisition of data; MFS, DC, CV, AL analysis and interpretation of data (eg, statistical analysis, biostatistics, computational analysis); MFS, GR, JK, DC, AL writing, review and/or revision of the manuscript.

## Data Availability

All data that were generated or analysed during this study are included in this published article (and its supplementary information files).
