# Supplementary material for: Anti‐human CD9 antibody Fab fragment impairs the internalization of extracellular vesicles and the nuclear transfer of their cargo proteins
Source: J Cell Mol Med. 2019 Apr 13;23(6):4408–21. doi: 10.1111/jcmm.14334 (PMC6533511; doi:10.1111/jcmm.14334)
Supplement: Supplementary file 1 [file JCMM-23-4408-s001.docx]

**Supplementary Information**

**
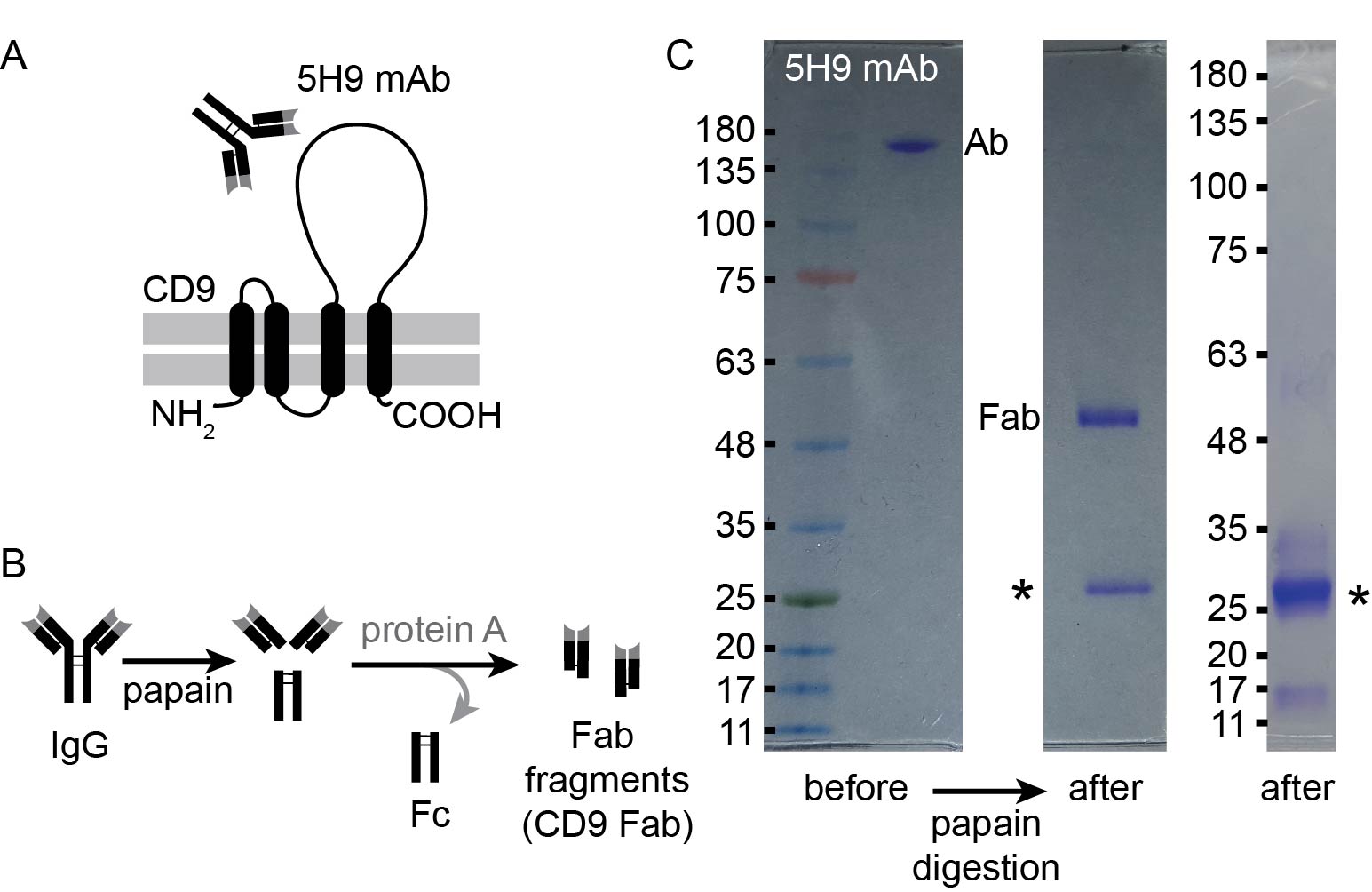
**

**Supplementary Figure 1.**

Generation of Fab fragment directed against CD9. (A, B) Schematic representations of 5H9 Ab (CD9 Ab) and its antigen, the tetraspanin CD9 (A), and the stepwise protocol for the generation of CD9 Fab, which includes a papain digestion of full-size Ab and the extraction of the derived Fc fragment by immobilized protein A (B). Under this setting, CD9 Fab is recovered in the flow through. (C) Full-size 5H9 Ab and Fab fragments thereof upon digestion by papain and extraction by protein A. Samples were resolved on SDS-PAGE under non-reducing (left lanes) and reducing (right lane) conditions and stained with Coomassie brilliant blue. Position of pre-stained molecular weight markers (kDa) are indicated. Ab, full-size Ab; Fab, Fab fragment; asterisk, digested and reduced Fab/light chain.

**
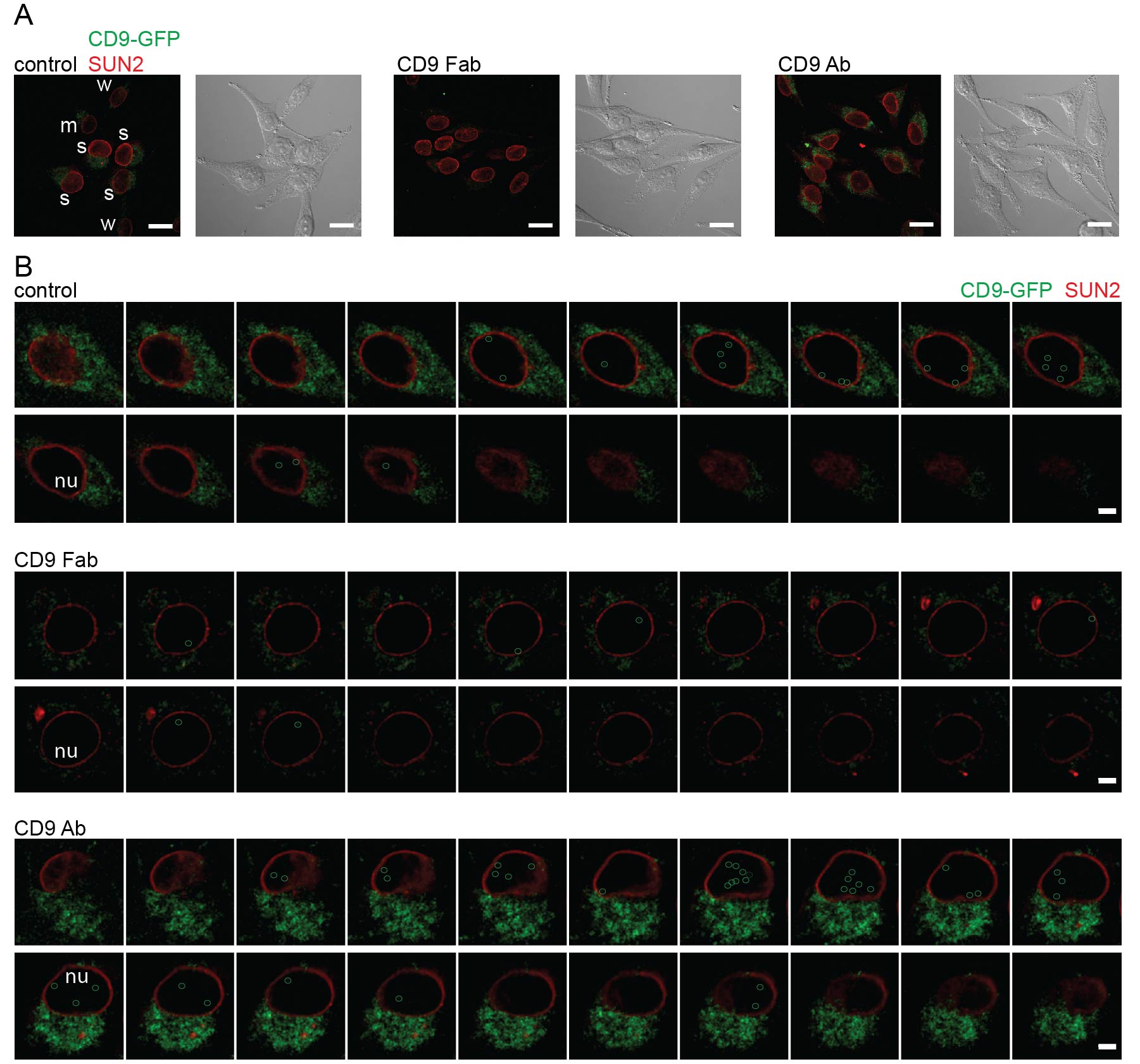
Supplementary Figure 2.**

CD9 antibodies influence the uptake of EVs and nuclear transfer of their cargo membrane proteins in melanoma cells. (A) FEMX-I cells were pre-incubated without (control) or with CD9 Fab or CD9 Ab (25 µg/ml) for 30 min and then exposed to CD9-GFP^+^ EVs (2.5 x 10^8^ particle/ml; green) for 5 hours. Afterward, they were immunolabeled for SUN2 (red) and analyzed by CLSM. Fluorescence and phase contrast micrographs are displayed at low magnification. In control, GFP fluorescence appears as strong (S), medium (M), or weak (W) among cells, while it becomes more homogenous upon the addition of antibodies. (B) Serial x-y optical sections (0.4-µm each) of FEMX-I cells exposed to antibodies and then to CD9-GFP^+^ EVs as described in panel A. Examples of cells used for the quantification of cytoplasmic and nuclear CD9-GFP signals using Fiji software are shown. Circles highlight the discrete, punctate CD9-GFP in the nucleoplasm (nu) of recipient cell. Note that they are often concentrated on few sections. Quantifications are presented in Fig. 3B and 3C. Scale bars, 5 µm.

**
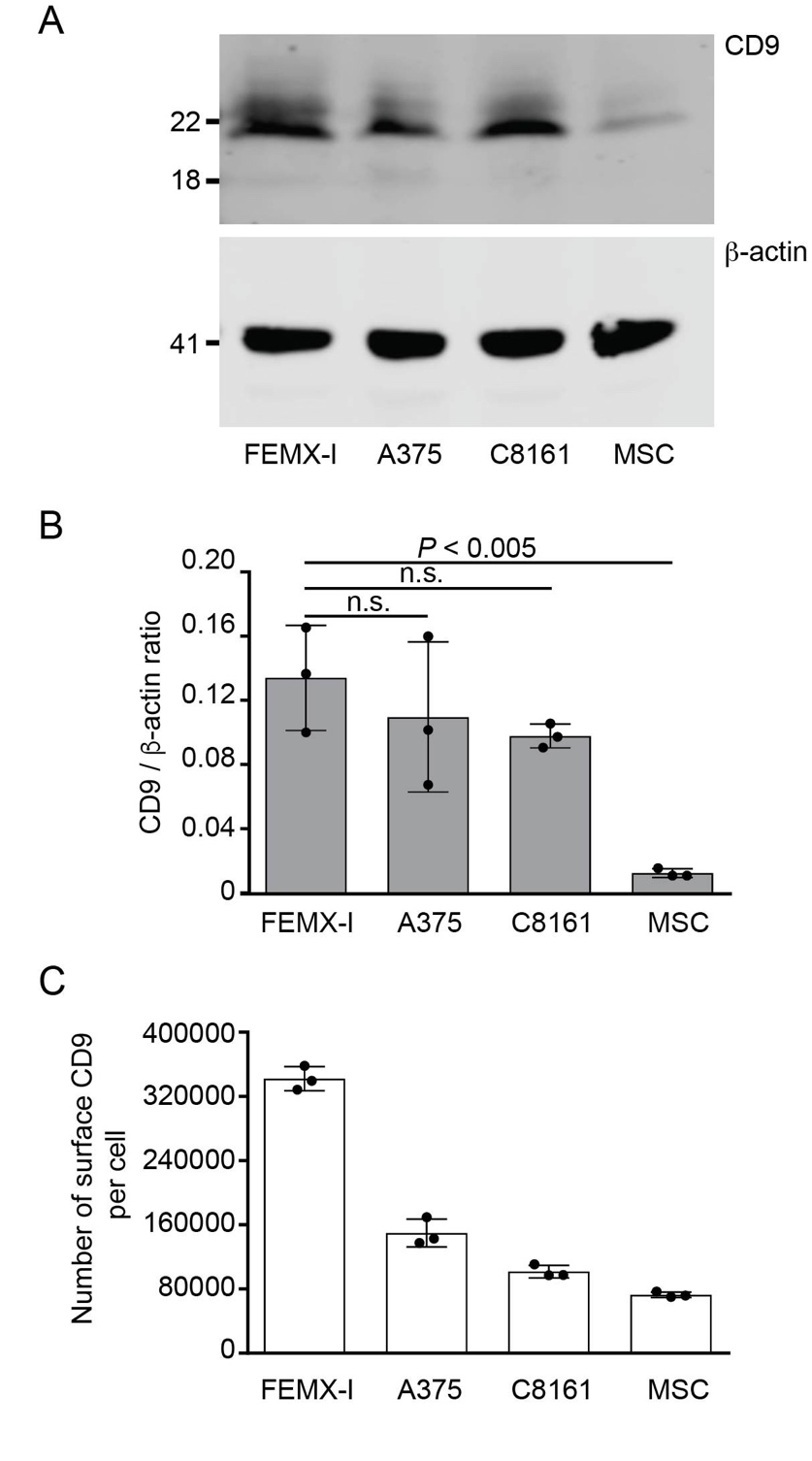
**

**Supplementary Figure 3.**

Quantification of CD9 in melanoma cell lines and primary MSCs. (A, B) Cell lysates prepared from melanoma FEMX-I, A375, and C8161 cells as well as MSCs were probed for CD9 and β-actin by immunoblotting. Molecular mass markers (kDa) are indicated. Representative blots are shown. (B) The ratio of CD9 to β-actin immunoreactivities were quantified (n = 3). (C) The amount of cell surface CD9 molecules per cell was quantified using QSC microsphere technology. Cells were labeled with PE-conjugated anti-CD9 Ab and analyzed by flow cytometry. The number of surface CD9 per cell was quantified using a standard curve (n = 3). In all cases, means ± S.D. are shown. *P-*values are indicated. N.s., not significant.
